# Current clinical spectrum of common variable immunodeficiency in Spain: The multicentric nationwide GTEM-SEMI-CVID registry

**DOI:** 10.3389/fimmu.2022.1033666

**Published:** 2022-10-28

**Authors:** Marta Dafne Cabañero-Navalon, Victor Garcia-Bustos, Maria Nuñez-Beltran, Pascual Císcar Fernández, Lourdes Mateu, Xavier Solanich, Juan Luis Carrillo-Linares, Ángel Robles-Marhuenda, Francesc Puchades-Gimeno, Ana Pelaez Ballesta, Nuria López-Osle, Miguel Ángel Torralba-Cabeza, Ana María Bielsa Masdeu, Jorge Diego Gil, Nuria Tornador Gaya, Guillem Pascual Castellanos, Rosario Sánchez-Martínez, José Manuel Barragán-Casas, Andrés González-García, José Luís Patier de la Peña, Daniel López-Wolf, Antonia Mora Rufete, Alba Canovas Mora, Maria José Forner Giner, Pedro Moral Moral

**Affiliations:** ^1^ Department of Internal Medicine, University and Polytechnic Hospital LaFe, Valencia, Spain; ^2^ Department of Internal Medicine, Germans Trias i Pujol University Hospital, Badalona, Spain; ^3^ Department of Internal Medicine, Bellvitge University Hospital, Barcelona, Spain; ^4^ Department of Internal Medicine, Virgen de la Victoria University Hospital, Málaga, Spain; ^5^ Department of Internal Medicine, La Paz University Hospital, Madrid, Madrid, Spain; ^6^ Department of Internal Medicine, University General Hospital of Valencia, Valencia, Spain; ^7^ Department of Internal Medicine, Rafael Méndez University Hospital, Murcia, Spain; ^8^ Department of Internal Medicine, Cruces University Hospital, Bizkaia, Spain; ^9^ Department of Internal Medicine, Lozano Blesa University Clinical Hospital, Zaragoza, Spain; ^10^ Department of Internal Medicine, Miguel Servet University Hospital, Zaragoza, Spain; ^11^ Department of Internal Medicine, University Hospital October 12, Madrid, Spain; ^12^ Department of Internal Medicine, University General Hospital of Castellón, Castellón, Spain; ^13^ Department of Internal Medicine, University General Hospital of Alicante, Alicante, Spain; ^14^ Department of Internal Medicine, Complejo Asistencial de Ávila, Ávila, Spain; ^15^ Department of Internal Medicine, Santiago Ramón y Cajal University Hospital, Madrid, Spain; ^16^ Department of Internal Medicine, University Hospital Alcorcón Foundation, Madrid, Spain; ^17^ Department of Internal Medicine, General University Hospital of Elche, Alicante, Spain; ^18^ Department of Internal Medicine, Clinical University Hospital of Valencia, Valencia, Spain

**Keywords:** common variable immunodeficiency, CVID, cohort, dysimmunity, immune dysregulation, lymphoproliferation

## Abstract

Common variable immunodeficiency (CVID) constitutes a heterogenic group of primary immunodeficiency disorders with a wide-ranging clinical spectrum. CVID-associated non-infectious morbidity constitutes a major challenge requiring a full understanding of its pathophysiology and its clinical importance and global variability, especially considering the broad clinical, genetic, and regional heterogeneity of CVID disorders. This work aimed to develop a nationwide, multicenter, retrospective study over a 3-year period describing epidemiological, clinical, laboratory, therapeutic, and prognostic features of 250 CVID patients in Spain. The mean diagnostic delay was around 10 years and most patients initially presented with infectious complications followed by non-infectious immune disorders. However, infectious diseases were not the main cause of morbimortality. Non-infectious lung disease was extraordinarily frequent in our registry affecting approximately 60% of the patients. More than one-third of the patients in our cohort showed lymphadenopathies and splenomegaly in their follow-up, and more than 33% presented immune cytopenias, especially Evans’ syndrome. Gastrointestinal disease was observed in more than 40% of the patients. Among biopsied organs in our cohort, benign lymphoproliferation was the principal histopathological alteration. Reaching 15.26%, the global prevalence of cancer in our registry was one of the highest reported to date, with non-Hodgkin B lymphoma being the most frequent. These data emphasize the importance of basic and translational research delving into the pathophysiological pathways involved in immune dysregulation and diffuse lymphocytic infiltration. This would reveal new tailored strategies to reduce immune complications, and the associated healthcare burden, and ensure a better quality of life for CVID patients.

## Introduction

Common variable immunodeficiency (CVID) constitutes a heterogenic group of primary immunodeficiency disorders (PID) with an estimated prevalence of 1:50.000 – 1:25.000 (1–3). It is characterized by decreased levels of serum IgG, together with decreased IgM and/or IgA, and it can be diagnosed after excluding secondary causes of hypogammaglobulinemia and other primary immunodeficiencies (1, 2). Infectious diseases were its main cause of morbidity and mortality until the introduction of immunoglobulin replacement therapy (IgRT) in the late 20^th^ century. Due to this treatment, non-infectious complications such as autoimmune, benign lymphoproliferative disorders, and even cancer have emerged as comorbidities with a larger impact on prognosis than infections (4). These dysimmune phenomena may affect up to 70% of patients (5, 6) and imply an 11-fold increased risk of death (5, 7). In fact, IgRT does not seem to prevent or improve many of these conditions (5).

Therefore, the clinical spectrum of CVID is wide-ranging. Back in 2008, Chapel et al. already described five clinical phenotypes of CVID: those that showed polyclonal lymphocytic infiltration, those with autoimmune diseases, those that developed enteropathy, those showing malignant neoplasia, and those only suffering infectious diseases (8). These non-infectious phenomena can be the first symptom of CVID disease in some patients (9) or can appear over time. Polyclonal infiltration may affect lymph nodes, liver, spleen, skin, or lung -causing the well-known granulomatous-lymphocytic interstitial lung disease (GLILD)- although every organ might be virtually infiltrated (10). The most common autoimmune complications are blood cytopenias. Nevertheless, almost every autoimmune disease has been described in association with CVID (7, 11). Some patients with CVID present with non-infectious inflammatory gastrointestinal disease resulting in diarrhea, malabsorption, and weight loss (12). Additionally, hematologic, and solid organ malignancies have been more frequently described in CVID than in the rest of PIDs and are associated with poorer outcomes (4, 13).

Thus, CVID-associated non-infectious morbidity constitutes a major challenge requiring a full understanding of its pathophysiology and its clinical importance and variability worldwide, especially considering the wide clinical, genetic, and even regional heterogeneity of CVID disorders. Moreover, many of these complications might be part of a common altered immunological substrate resulting in a complex multisystemic disease that needs to be further characterized (14). The development of standardized targeted therapies is also required, as IgRT only plays a role in preventing infectious disorders. The use of other anti-inflammatory or immunosuppressant agents for immune complications is not exempt from serious adverse effects in these already immunocompromised patients and lacks a wide body of evidence (15).

Consequently, registries and large cohort studies are needed in order to provide an updated epidemiological overview of the clinical spectrum of CVID with less biased results considering high variability and even regional diversity of these disorders. The aim of this work was to develop a nationwide, multicenter, retrospective study over a 3-year period describing epidemiological, clinical, laboratory, therapeutic, and prognostic features of CVID patients in Spain.

## Material and methods

### Study design, setting, and population

A multicenter, cross-sectional, nationwide study of patients diagnosed with CVID was carried out in Spain from November 2019 to May 2022. A total of 17 hospitals treating PIDs participated in the creation of the GTEM-SEMI-CVID-Registry promoted by the Working Group of Minority Diseases of the Spanish Society of Internal Medicine (GTEM-SEMI). All patients aged 16 years and above with CVID diagnosis according to the European Society for Immunodeficiencies (ESID) registry working definitions (2) and who were or had been followed up by the participating units were considered eligible. By protocol, all selected patients for inclusion were initially screened to confirm CVID diagnosis according to the consensus ESID criteria, and patients with definite monogenic immunodeficiencies were not eligible for inclusion at the time of data collection.

### Data collection and variables

The GTEM-SEMI-CVID-Registry systematically compiles sociodemographic and epidemiological data, genetic information, infectious and non-infectious comorbidities, imaging findings, laboratory and histopathological parameters, treatments, and patient outcomes. All data were collected by reviewing electronic medical records and included in a predesigned database.

Demographic data included sex, current age, or age at the time of death, age at diagnosis, age at clinical onset, diagnostic delay, first PID-related clinical complication (infection, dysimmunity, malignancy, or other), and time of follow-up. Genetic testing, family history, and consanguinity were recorded. All described genes with variants not listed under CVID and attributable to defined monogenic PIDs were compulsorily those of ‘uncertain significance’ or ‘likely benign’, according to the Standards and Guidelines for the Interpretation of Sequence Variants of the American College of Medical Genetics and Genomics and the Association for Molecular Pathology.

History of major bacterial infections, opportunistic, and recurrent infections were collected, including chronic Epstein-Barr virus (EBV) and *Helicobacter pylori* infections. Non-infectious comorbidities included autoimmune cytopenias, lymphadenopathies, splenomegaly, hepatomegaly, and clinical or imaging findings of portal hypertension, systemic autoimmune disorders, lung, gastrointestinal, cutaneous, and neurological involvement, as well as cardiovascular comorbidity and malignancy (both solid and hematological neoplasia). Furthermore, clinical symptoms such as chronic cough, dyspnea, chronic diarrhea, and abdominal pain were recorded. Chronic enteropathy was considered, if the presence of chronic diarrhea, and/or diagnosed malabsorption were recorded in absence of documented infection.

Imaging findings were limited to chest CT scanning due to frequent and potentially severe lung involvement in CVID and according to the follow-up recommendations of the British Society for Immunology/United Kingdom Primary Immunodeficiency Network Consensus on managing non-infectious complications of CVID (16). Additionally, and following previous recommendations, lung function tests (LFT) and values of corrected diffusing capacity of the lungs for carbon monoxide (cDLCO) were also considered.

Laboratory variables included IgG (mg/dL), IgM (mg/dL), IgA (mg/dL), CD4 cell count (cell/µL), CD8 cell count (cell/µL), CD4/CD8 ratio, CD19 cell count (cell/µL), CD3 cell count (cell/µL), and natural killers (NK) cell count (cell/µL) both at diagnosis and last determination after treatment, first available LDL (mg/dL), HDL (mg/dL), triglycerides levels (mg/dL), positivity at any point of autoantibodies such as antinuclear antibodies (ANA) -only if titres exceed 1/80-, extractable nuclear antigens (ENA), rheumatoid factor (RF), antineutrophil cytoplasmic antibodies (ANCA), and myositis-related and systemic sclerosis-related antibodies. Due to the high prevalence of bowel disease, positivity at any point of coeliac-related antibodies, intrinsic factor, and gastric parietal cells antibodies (GPC), and HLA DQ2/DQ8 typing were also noted.

Histopathological results of lymph nodes, spleen, liver, lung, and digestive tract were compiled. In lymph node and spleen biopsies, the presence of granulomatosis, benign lymphoproliferation, lymphoma, or other features were collected. In liver biopsies, evidence of fibrosis or nodular regenerative hyperplasia (NRH) was registered too. For those patients who underwent lung biopsy, histologically relevant findings were classified as lymphoid interstitial pneumonia (LIP), GLILD, follicular bronchiolitis, granulomas, lymphoma, and their combinations. Gastric and bowel histology was comprehensively studied, including variables such as atrophic gastritis, nodular lymphoid hyperplasia (NLH), intestinal villous atrophy, lymphocytic infiltration, eosinophilic infiltration, absence of plasma cells, presence of granulomas, lymphocytic colitis, or findings compatible with Crohn’s disease or ulcerative colitis.

Treatment with IgRT, regime, and route of administration were also included. Moreover, therapy with all categories of immunosuppressant agents received at any point of the clinical history was considered. The GTEM-SEMI-CVID-Registry also collected the mortality rate, cause of death, and follow-up time until death in those deceased patients.

### Statistics

Owing to the epidemiological and cross-sectional nature of the study, variables were mainly analyzed using descriptive statistics. The analyses were performed with R statistical software version 4.2.1 (R Development Core Team, 2022). Quantitative data were expressed as mean and standard deviation (SD). Qualitative data were expressed as absolute count and percentage of cases, without accounting for non available values. For subanalyses of variables of interest, significance was assessed with the χ2 or Fisher exact test for categorical variables and the Student t test for continuous variables after checking pertinent statistical assumptions. Two-tailed p-value below 0.05 was considered statistically significant.

### Ethical statement

The creation and protocol of the GTEM-SEMI-CVID-Registry were independently approved by all institutional Ethical Committees of each participating hospital under their corresponding registry codes. Anonymity and data confidentiality of all included patients were ensured in accordance with the Spanish regulation of observational studies. The work was performed according to the Declaration of Helsinki and followed the STROBE guidelines (**Supplementary Material**).

## Results

### Demographics

As of May 2022, a total of 250 patients diagnosed with CVID were included in the GTEM-SEMI-CVID Spanish Registry. In our cohort, the mean age was 54.08 (SD 18.22) years old, and 121 patients (48.4%) were male. The mean age at diagnosis was 40.89 (SD 19.74) years old. However, the mean diagnostic delay was 10.01 (SD 13.21) years, while the global mean age at characteristic symptom onset was 29.8 (SD 20.08) years old. Most patients initially presented with infectious complications (158, 63.2%), followed by non-infectious or neoplastic complications (83, 33.2%). Two patients presented with non-Hodgkin lymphoma. Family history and consanguinity were registered in 226 and 193 patients, respectively. Twenty-seven (11.95%) patients had a previous family history of CVID. History of consanguinity was seen in 8 patients (4.14%). Genetic testing was performed in 99 (39.6%) patients. Genetic variants were found in 35 patients, and all of them were considered benign or of unknown significance (**Table 1**).

**Table 1:** List of genetic variants in the Spanish GTEM-SEMI-CVID Registry.

Genetic variants	N
*TACI*	7
*NFKB1*	7
*CTLA4*	5
*MBL2*	3
*IKAROS*	2
*BTK*	1
*NFKB2*	1
*LRBA*	1
*MLL2*	1
*PI3KCD*	1
*PI3KR1*	1
*PCLG2*	1
*PTPN2*	1
*RAG1*	1
*TFC3*	1
*CD27*	1

N, number of patients with mutations in the mentioned gene.

### Infectious complications

Information on the history of infectious complications of CVID was available for all patients included in the cohort, with airway infections being the most common. Almost 65% had suffered at least one episode of a major bacterial infection, and pneumonia accounted for most episodes. A history of sepsis, nevertheless, was only reported in 31 patients. Of all respiratory infections, more than 50% were recurrent and around 70% of patients reported a previous history of recurrent upper respiratory tract infections. Skin, soft tissue, or musculoskeletal infections were less frequent than opportunistic infections (considering deep-seated candidiasis, cryptococcosis, tuberculosis (TBC), and infection by non-TBC mycobacteria, *Pneumocystis jirovecii* pneumonia, cytomegalovirus (CMV) infection, JC virus infection, leishmaniasis or toxoplasmosis among others). Noteworthy, there were 2 reported cases of TBC, 3 cases of disseminated CMV infection, and 1 case of visceral leishmaniasis. Further information is detailed in **Table 2**.

**Table 2:** Descriptive statistics of the major bacterial and the recurrent infections in the GTEM-SEMI-CVID Registry.

	N	Frequency (%)
**Major bacterial infections**		
Major bacterial infections	159	63.6
Abdominal infections	29	11.6
Cellulitis	20	8
Febrile UTI	17	6.8
Meningitis	8	3.2
Opportunistic infections	24	9.64
Osteomyelitis	2	0.8
Pneumonia	146	58.5
Sepsis	31	12.4
**Recurrent infections**		
Upper airway	178	70.8
Lower airway	133	53.2
Gastrointestinal	59	23.6
Urinary	21	8.4
Intestinal parasitosis	23	9.2
SST	11	4.4

N, number of patients with the mentioned variable; SST, soft and skin tissue infections; UTI, urinary tract infections.

A significantly higher proportion of patients who suffered opportunistic infections had received or were under treatment with corticosteroids (68.2% versus 32.2%, χ2 p=0.002). Additionally, this was also significantly observed when considering treatment with azathioprine (31.8% versus 10.3%, χ2 p=0.01), as well as the combination of corticosteroids, azathioprine, and rituximab (20.8% versus 5.3%, χ2 p=0.015). No other significant differences were observed in relation to rituximab alone or other immunosuppressive treatments, or in relation to CD4+ cell count and genetic mutations. However, the genetic information is limited due to the absence of genetic analysis in a large part of the studied population.

### Overview of non-infectious immune complications

**Figure 1:**
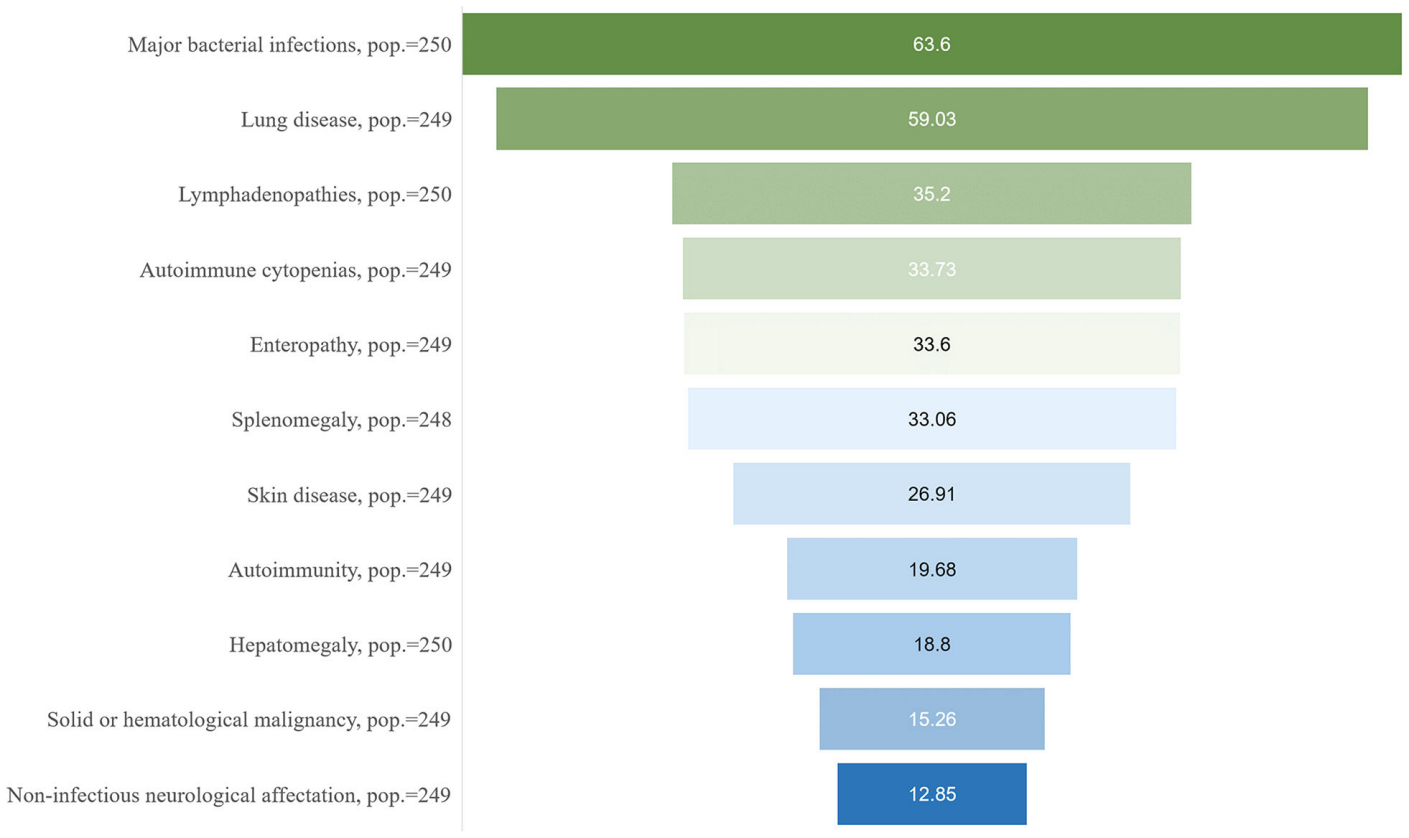
Summary of major comorbidities of the CVID patients included in the GTEM-SEMI-CVID Registry. Pop., number of patients for whom information on the specified variable was available.

Forty-three patients showed non-infectious immune complications as the first manifestation of the disease. During the mean follow-up time until data collection (8.90 years, SD 7.90), the most common immune complication was the presence of lymphadenopathies (35.2%), closely followed by the development of immune cytopenias (33.73%), non-infectious enteropathy (33.60%) and splenomegaly (33.06%). A total of 19.68% of patients had been previously diagnosed with a systemic autoimmune disease. Liver disease was less frequent, with hepatomegaly and liver nodules being found in 18.8% and 6.5% of patients, respectively (**Figure 1**). Considering GLILD as a severe lung immune complication in CVID, its presence was histologically confirmed in 7.69% of patients. Dysimmune neurological complications were also reported in 15 patients (6.02%) of the cohort.

Organ-specific manifestations will be described in detail below.

### Systemic autoimmune diseases and immunosuppressant drugs

Cytopenias were present in 84 patients of the cohort. Two hundred and thirty-one patients were recorded for the specific presence of cytopenias. Evans’ syndrome, defined as both the simultaneous presence of immune anemia and thrombopenia, as well as leukopenia and thrombopenia were the most frequent cytopenias in our cohort, reported in 12 and 11 patients, respectively (5.19% and 4.76%). Isolated immune thrombopenia and hemolytic anemia were present in 6 (2.60%) and 4 (1.73%) patients, respectively. Isolated immune neutropenia was reported in 4 patients (1.73%).

**Figure 2:**
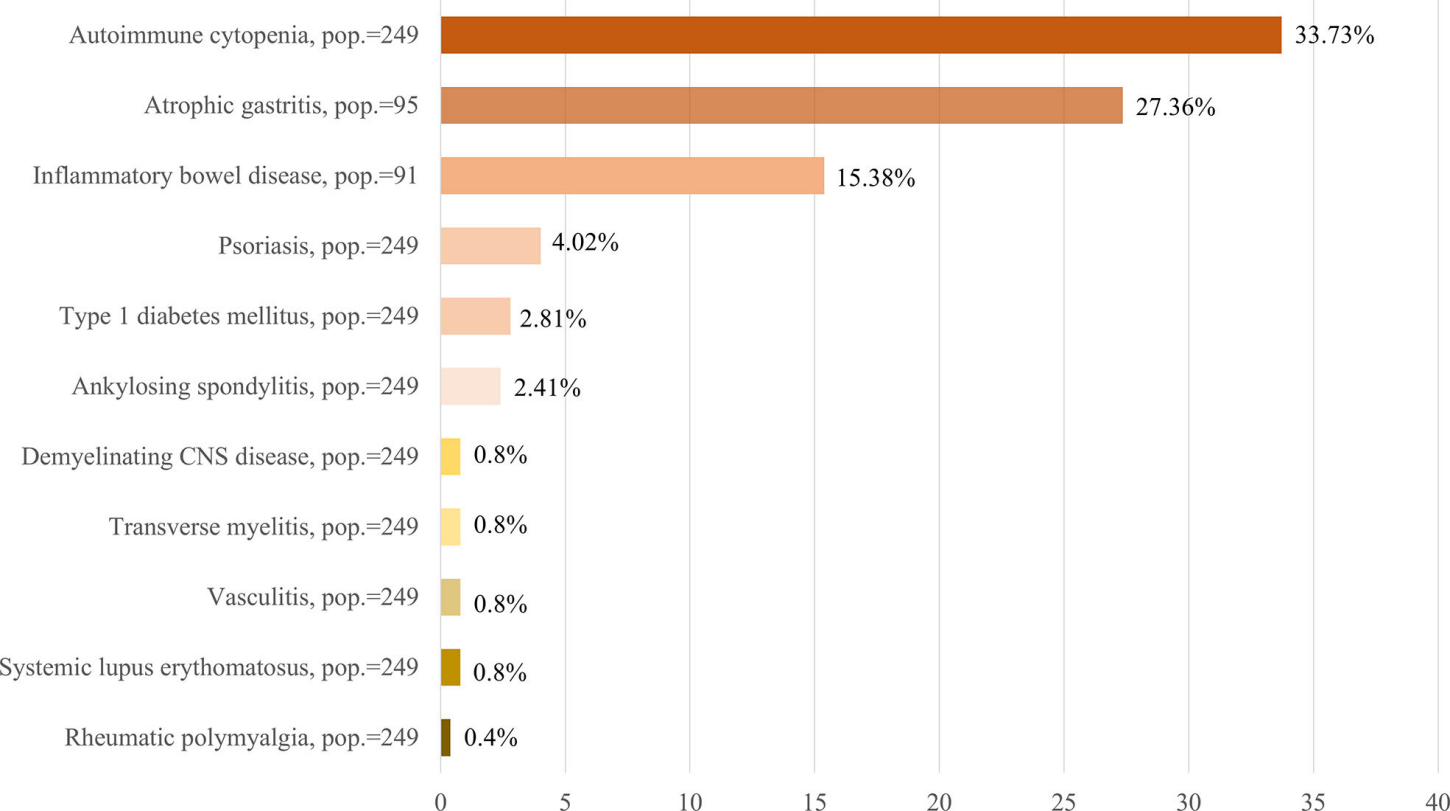
Summary of main immune complications of the CVID patients included in the GTEM-SEMI-CVID Registry. CNS, central nervous system. Pop., number of patients for whom information on the specified variable was available.

Autoimmunity was present in 49 patients (19.68%) (**Figure 1**). The most common rheumatic disease in our cohort was ankylosing spondylitis, with a prevalence of 2.41% (6 cases), closely followed by sarcoidosis (2% and 5 cases). Systemic lupus erythematosus (SLE) and unspecified vasculitis had been diagnosed in 2 patients each (0.80%), and only 1 case of Sjögren syndrome and rheumatic polymyalgia were described (**Figure 2**). No reports on other autoimmune diseases such as systemic sclerosis or rheumatoid arthritis were seen. Type 1 diabetes mellitus was reported in 7 patients (2.81%) (**Figure 2**). Antinuclear antibodies (ANA), extractable nuclear antigen antibodies (ENA) and anti-neutrophil cytoplasmic antibodies (ANCA) were positive in 8.84% (17), 1.77% (2), and 2.86% (4) of determinations. Of all patients, 94 had received pharmacological immunosuppressant or immunomodulatory treatments (**Table 3**). Corticosteroids (34.17%), followed by azathioprine (12.24%) and rituximab (12.23%) were the most frequently used immunosuppressant agents, mainly targeting CVID immune complications such as cytopenias, lymphoproliferation, and autoimmune disorders

**Table 3:** History of immunosupressant treatment in the GTEM-SEMI-CVID Registry.

	N	Frequency (%)
Patients who received immunosuppressant therapies	94	38.68
Corticosteroid treatment	81	34.17
Antimalarial treatment	6	2.55
Abatacept	1	0.42
Anakinra	0	0
Azathioprine	29	12.24
Belimumab	0	0
Cyclophosphamide	1	0.42
Cyclosporin	2	0.84
Etanercept	0	0
Infliximab	5	2.12
Mofetil mycophenolate	9	3.81
Methotrexate	2	0.84
Rituximab	29	12.23
Sirolimus	2	0.84
Tacrolimus	7	2.96

N, number of patients that received each treatment.

Eighty-one patients had received or were under corticosteroid treatment at the time of inclusion, of which 52 presented autoimmune cytopenias, 14 had histologically confirmed GLILD, and 24 patients suffered intestinal malabsorption. Azathioprine had been administered in 21 patients affected by immune cytopenias and 12 patients with confirmed GLILD. Rituximab therapy was the most used monoclonal antibody, and it had been administered in 24 patients with immune cytopenias, 14 patients with GLILD and in 6 patients who suffered non-Hodgkin lymphoma. Seventeen patients received treatment with all three agents, of which 10 patients presented coexistence of autoimmune cytopenias and GLILD. Further information on significant immunosuppressant therapies and the observed comorbidities in these patients can be consulted in the **Supplementary S1 File**.

### Spleen and lymph node involvement

The presence of significant lymphadenopathies was observed in 88 patients. Half of them (18) had undergone a lymph node biopsy. Eighty-two patients (33.06%) presented splenomegaly (**Figure 1**), but a splenic biopsy was only performed in 4 patients due to high hemorrhagic risk. Benign lymphoproliferation (65.91% of lymph node biopsies and 50% of spleen biopsies) and node granulomatosis (44.45% of lymph node biopsies and 75% of spleen biopsies) were the most frequent histological findings for both organs. Further histological data are detailed in **Table 4**.

**Table 4:** Lymph node, spleen, liver, lung, and gastrointestinal histopathology of GTEM-SEMI-CVID Registry.

	N	Frequency (%)
**Lymph node biopsy**	44	17.89
Node granulomatosis	20	44.45
Node lymphoma	9	20.45
Node benign lymphoproliferation	29	65.91
Other	1	2.27
**Spleen biopsy**	4	1.6
Spleen granulomatosis	3	75
Spleen lymphoma	1	25
Spleen benign lymphoproliferation	2	50
Other	1	25
**Liver biopsy**	12	4.8
Liver granulomatosis	5	41.67
Liver fibrosis	3	25
Liver benign lymphoproliferation	1	8.33
Liver nodular hyperplasia	7	58.33
**Lung biopsy**	31	13.06
Follicular bronchiolitis	2	6.45
Granulomatosis	1	3.22
Advanced interstitial pneumonia	1	3.22
Lymphocytic interstitial pneumonia	7	22.58
Lymphocytic interstitial pneumonia and bronchiolitis	1	3.22
Lymphocytic interstitial pneumonia and granulomatosis	8	25.81
Other	13	41.94
**Diagnosed GLILD**	17	7.69
**Gastroduodenal biopsy**	95	38.61
**Small intestine biopsy**	49	20.16
**Colon biopsy**	76	31.27
**Histological findings**		
Atrophic gastritis	26	27.36
Gastrointestinal nodular lymphoid hyperplasia	38	40
Inflammatory bowel disease	14	15.38
Intestinal eosinophilic infiltrate	9	9.47
Intestinal plasmatic cell absence	35	39.66
Intestinal granulomatosis	3	3.37
Intestinal lymphocytic infiltrate	37	38.95
Lymphocytic colitis	16	18.39
Villous atrophy	26	27.08

### Liver involvement

In 47 patients (18.8%), hepatomegaly was documented on imaging tests regardless of the presence of splenomegaly. Liver nodules were observed in 16 patients (6.5%). Clinical, laboratory, imaging, or endoscopic evidence of portal hypertension was present in 19 patients (7.75%), and liver biopsy had been performed in 12 individuals (4.8%) during follow-up. As seen in **Table 4**, liver nodular hyperplasia was the most frequent histological finding (58.33%) followed by liver granulomatosis (41.67%).

### Lung involvement

Non-infectious lung disease was extraordinarily frequent and was seen in almost 60% of CVID patients (**Figure 1**). Nevertheless, less than 25% of them reported chronic dyspnea or cough as the most frequent clinical manifestation (**Table 5**). High-resolution CT (HRCT) scanning had been performed for nearly 65% of the patients and isolated bronchial affectation, mainly bronchiectasis was the most frequent imaging finding (35%). Lung parenchymal involvement was observed in 15% of the patients, and the most regularly described radiological pattern was compatible with lymphoid interstitial pneumonia (13.83% of HRCT scans). Almost 80% of the patients had undergone LFT during follow-ups. Normal results were obtained in 56.90% of them and an obstructive spirometry pattern was seen in 27.59%. The mean cDLCO was 73.62 (21.28 SD). Further data are detailed in **Table 5**. Thirty-one patients underwent lung biopsy. Histological results concurred with imaging observations, and combined lymphocytic interstitial pneumonia and granulomatosis were the most frequent findings (25.81% of lung biopsies), as seen in **Table 4**. Confirmed GLILD according to the British Lung Foundation Criteria (15) was seen in 17 patients (**Table 5**).

**Table 5:** Lung disease in the GTEM-SEMI-CVID Registry.

	N	Frequency (%)
**Lung disease**	147	59.03
**Dyspnoea**	48	19.91
**Cough**	56	23.14
**Chest HRCT performed**	158	64.49
Normal	67	33.33
Bronchial	72	35.82
Parenchymatous	31	15.42
Bronchial and parenchymatous	24	11.94
Bronchial, parenchymal and pleural	2	1
Bronchial and pleural	2	1
Parenchymal and pleural	3	1.49
**Radiological patterns**		
Respiratory bronchiolitis and interstitial lung disease	1	0.53
Desquamative interstitial pneumonia	1	0.53
Lymphocytic interstitial pneumonia	26	13.83
Non-specific interstitial pneumonia	10	5.32
Usual interstitial pneumonia	1	0.53
Normal	149	79.26
**Lung function test (LFT)**	188	79.66
**LFT pattern**		
Normal	99	56.9
Obstructive	48	27.59
Restrictive	13	7.47
Mixed	14	8.05
**Diagnosed GLILD**	17	7.69

GLILD, granulomatous-lymphocytic interstitial lung disease; HRCT, high resolution computerized tomography; LFT, lung function test; N, number of patients with the mentioned variable

### Digestive tract involvement

Gastrointestinal affectation was common (40.56%) but also highly variable. Non-infectious diarrhea was reported in 83 patients (33.60%) (**Figure 1**). However, proved malabsorption was diagnosed in 30 patients (23.81%). Determination of H. pylori infection was performed in 126 patients, with a prevalence in our population of 23.81%. Screening for coeliac, anti-gastric parietal, and anti-intrinsic factor antibodies, as well as screening for HLA DQ2/DQ8, was carried out in 241, 71, 53, and 33 patients obtaining a positive result in 3.73%, 19.72%, 3.77%, and 24.24%, respectively. Gastroduodenal, small intestine, and colon biopsies were performed in 95, 49, and 76 patients, correspondingly. Histological findings are detailed in **Table 4**. To highlight, lymphoid nodular hyperplasia, lymphocytic infiltrate, and intestinal absence of plasma cells were the most common traits. Atrophic gastritis was observed in 27.36% of the patients (**Figure 2**).

### Involvement of other organs: cutaneous and neurological manifestations

Skin disorders were reported in 67 patients in our cohort (26.91%) (**Figure 1**). The most frequent lesions were eczema, in 22 patients (8.83%); psoriasis, in 10 patients (4.02%) (**Figure 2**); followed by vitiligo and alopecia, both recorded for 6 patients (2.41%). The presence of warts was registered for 3 patients (1.20%). However, the occurrence of other non-specified lesions of a wide nature (cutaneous neoplasms, nevi, among others) was reported in 37 individuals.

The presence of non-infectious neurological involvement was also studied. Five patients (2.01%) presented both intellectual disability and peripheral neuropathy of diverse severity and etiology. Of note, 4 patients suffered epilepsy (1.60%) and immune demyelinating lesions and transverse myelitis were described in 2 cases each (**Figure 2**). One episode of subacute sclerosing panencephalitis with severe sequelae was reported after measles vaccination in an undiagnosed CVID patient at that moment.

### Neoplastic complications

Prevalence of both solid and hematological malignancies from inclusion to follow-up was 15.26% (38 cases) (**Figure 1**). Non-Hodgkin B lymphoma (11 cases and 4.41%) was the most frequent malignancy in our population. Gastric cancer and lung adenocarcinoma were reported in 5 (2.01%) and 3 (1.20%) patients, respectively. Interestingly, only 2 cases of colorectal cancer, as well as 1 case of breast cancer and prostate cancer were diagnosed. Of note, 3 patients reported basal cell carcinomas, and 1 patient was diagnosed with both basal cell and skin squamous cell carcinoma of the skin. The remaining 7 cases were diagnosed with other less frequent tumors (namely, myeloma, splenic lymphoma, thyroid, renal, uterine, or cervical cancer).

### Other comorbidities

The prevalence of diseases resulting in increased cardiovascular events such as hypertension, type 2 diabetes mellitus (DM), dyslipidemia, and gout were seen in 52, 22, 41, and 4 individuals (namely, 20.88%, 8.84%, 16.65%, and 1.61%). Eighteen patients (7.23%) presented heart failure, 5 (2%) had suffered a stroke, and 3 (1.20%) reported peripheral arteriopathy. Chronic kidney disease was documented in 12 patients (4.82%). Venous thromboembolic disease was reported in 7 patients (2.81%).

### Immunological parameters

Immunoglobulin levels at diagnosis as well as lymphocyte subpopulation cell count are detailed in **Table 6**. There was wide variation in the immunoglobulin levels and B and T cell subpopulations cell counts.

**Table 6:** Data on mmunoglobulin levels and lymphocyte subpopulations count in the GTEM-SEMI-CVID Registry.

	Mean	Median	SD	Interquartile range
Serum IgG levels at diagnosis (mg/dL)	403.219	382	218.65	290.25
Serum IgA levels at diagnosis (mg/dL)	47.09	15	78.26	51.83
Serum IgM levels at diagnosis (mg/dL)	53.3	26	88.88	54.25
Total lymphocyte count at diagnosis (cell/μL)	1794.05	1500	1055.95	966.5
CD3 cell count at diagnosis (cell/μL)	1302.81	1151	708.06	774
CD4 cell count at diagnosis (cell/μL)	678.9	616	427.81	436
CD8 cell count at diagnosis (cell/μL)	520.6	432	393	453
CD19 cell count at diagnosis (cell/μL)	231.22	178	192.1	192.75

SD, standard deviation.

### IgRT and mortality

Two hundred and fourteen patients were under active treatment with IgRT. Remarkably, 29 patients were not being treated or had received IgRT at the time of data collection, and 3/29had already died. Therapeutic information was lacking for 8 patients in the national cohort. No differences were observed in the baseline epidemiological features of patients in the non-treated group, being only discretely younger than the treated group (46.10 vs 51.51 years). Intravenous route (IVIgRT) was preferred (124 patients, 57.94%) over subcutaneous (SCIgRT) (88, 41.12%) or intramuscular (IMIgRT) (1, 0.47%) routes. All patients received IVIgRT at the hospital, and most patients under SCIgRT (74 patients) administered it at home, or both (5).

Fifteen patients had died until the time of data collection, meaning a mortality rate of 6% in our cohort until data cut-off, with a mean age at death of 57 years old (SD 20.16) and a follow-up time until death of 7.6 years (SD 7.75). Non-infectious complications such as chronic respiratory failure (3 patients), solid or hematological malignancies (3 patients), or dysimmune complications (3 patients) were more frequent than infectious complications (3 patients) when analyzing the cause of death.

## Discussion

The GTEM-SEMI-CVID-Registry systematically compiles the current clinical profile of 250 patients diagnosed with CVID in Spain on the basis of a nationwide multicentric approach. Hence, a complete, updated, and integrated view of the CVID clinical course is detailed in this study, which increases the available data provided by other CVID cohorts.

In line with previous reports, the mean diagnostic delay in our study was around 10 years (8, 19, 20), and most patients initially presented with infectious complications followed by non-infectious immune disorders. However, the current diagnostic delay appears higher than previously reported in partial cohorts in our country, which ranged from 4-6 years (4). More than one-third of the patients in our cohort showed lymphadenopathies and splenomegaly in their follow-up, and more than 30% presented immune cytopenias, with a higher prevalence than in other international cohorts, possibly due to a relatively higher mean age of our patients and longer disease evolution (8, 14, 17, 19) (**Figures 1**, **2**). Remarkably, Evans’ syndrome was the most frequently described immune cytopenia instead of isolated ITP as previously described (5, 8, 14). Furthermore, systemic autoimmunity was reported in 20% of the patients, with anchylosing spondylitis being the most frequent rheumatic disease. This highlights the present need for developing referral protocols allowing an earlier diagnosis, which should include not only repeated infections as the main red flag for suspecting CVID and other immunodeficiencies (21, 22) but also immune disorders and organ lymphoproliferation.

Since the introduction of IgRT, infectious complications are not the main cause of morbimortality in CVID patients (5), as illustrated in this series. For more than a decade, SCIgRT has been introduced as a successful alternative to conventional IVIgRT with optimal IgG trough levels, fewer side effects, better health-related quality of life, patient empowerment, and faster functional recovery with less contact with the healthcare environment (23). Nevertheless, less than half of the patients received SCIgRT and IVIgRT was preferred by almost 60%. Through these data, practitioners should be encouraged to consider switching IVIgRT to SCIgRT for qualified patients with CVID.

Non-infectious lung disease was extraordinarily frequent in our registry affecting approximately 60% of the patients, which shows a higher prevalence than previously known (5, 8, 14) (**Figure 1**). Bronchiectasis was the most frequent imaging finding (35%) in consonance with previous studies (14). This could be due to chronic and repeated infections rather than local immune dysregulation (24). However, lung parenchymal involvement was observed in around 15% of the patients, mainly as lymphoid interstitial pneumonia. Despite the high prevalence of lung disease, less than 65% of the patients included in the cohort underwent a HRCT. This advocates that some patients with subclinical affectation may have been underdiagnosed in our cohort, as only around 20% experienced respiratory clinical manifestations such as cough or dyspnea. In line with this, histologically confirmed GLILD was only seen in 17 patients; a notably lesser prevalence than expected according to previous evidence (25, 26). This emphasizes the need for a further active search for chronic lung disease in the follow-up of these patients despite being asymptomatic or paucisymptomatic, as it has been associated with poorer clinical outcomes (8, 27).

In the GTEM-SEMI-CVID Registry, gastrointestinal disease was observed in more than 40%, which is the high end of previously reported data (range: 9 – 44%) (8, 14, 17, 19, 20, 28). Intestinal lymphocytic infiltration was the most common histological finding, being present in almost 40% of the biopsies, as recently published (14).

These results concur with growing evidence supporting disseminated benign lymphoproliferation and infiltration as one of the main characteristics of abnormal immunity in CVID patients, which may result in organ dysfunction at different levels. Among biopsied organs in our cohort, benign lymphoproliferation was the principal histopathological alteration, except for spleen and liver, where granulomatosis was observed in 75% and 42% of the biopsies, respectively. Interestingly, in non-lymphoid organs, lymphocytic infiltration was more frequent in gastrointestinal tract, probably due to the continuous antigen-lymphocyte interaction with local microbiota. Many studies have shown increased permeability of the gut barrier which results in microbial translocation and local inflammation, both endpoints of the systemic immune activation that was expressed in the histological findings of the patients in our cohort (29–31).

This coexistence of immunodeficiency and immune dysregulation with diffuse lymphocytic infiltration and autoimmune disorders in CVID patients seems paradoxical, requiring the use of immunosuppressant treatments on already immunosuppressed patients. In our case, almost 40% of the individuals had received immunosuppressant therapies, mainly corticosteroids followed by azathioprine and, notably, rituximab. These agents were mainly used to treat immune cytopenias, granulomatous or lymphocytic disorders, and other autoimmune complications. Given their risk of adverse effects, the need for these therapies reveals an important therapeutic complexity (32), especially considering the relatively high prevalence of opportunistic infections in our cohort, of up to 9.64% with clear relation to some of the most frequently used treatments. Further pathophysiological studies are needed to unravel the genetic and molecular pathways driving CVID dysimmunity that will allow the development of targeted therapies with fewer side effects.

In this regard, the presence of persistent abnormalities in immune activation and systemic inflammation have been related to an increased risk of cardiovascular disease (33, 34). Although the chronic proinflammatory state of CVID could be related to higher cardiovascular risk, evidence is still scarce (35–37). Nonetheless, the prevalence of cardiovascular comorbidities was not increased in our series when compared to the Spanish general population of the same age group. For instance, hypertension and type 2 DM are found in 42.6% and 14.8% of the Spanish adult population respectively, whereas in our study, the global prevalence of hypertension and type 2 DM was 20.88% and 8.84%, respectively. Chronic kidney disease (CKD) accounted for 4.82% of the patients in our registry, while the global prevalence of CKD is 15.1% for the Spanish adult population (38, 39). Studies aiming to profile the cardiovascular risk in CVID are needed to overcome this large knowledge gap.

Moreover, in the literature, patients with CVID have a significantly higher risk of both hematological and solid malignancies, with a described prevalence of approximately 10% (ranging from 1.5 – 20.7%). The overall prevalence of cancer in our registry was one of the highest reported to date, of up to 15.26% (5, 13, 14, 39–42) (**Figure 1**). As with immune complications, the higher mean age in the Registry as well as the diagnostic delay could partially explain these findings (13). In line with previous evidence, non-Hodgkin B lymphoma was the most frequently reported malignancy, followed by gastric cancer, lung tumors, and cutaneous malignancies. Despite the declining incidence of gastric cancer in older reports (43, 44), the prevalence of 2% in this series is significantly higher compared to the other recent studies (5, 14). The fact that approximately half of all patients had been actively investigated for the presence of H. pylori may have contributed to these higher figures and indicates the need for periodical screening for this infection in all CVID patients.

Several important limitations deserve mention. Firstly, this is a retrospective cohort study. Despite a systematic and closed form with clear instructions and definition of variables was provided, data were collected by a large number of researchers which could have led to heterogeneity in data input and validation. In fact, due to the retrospective approach based on the review of electronic clinical records, some information was not available, thus limiting its extrapolation to the general population with CVID. Secondly, despite the large number of clinical and complementary variables included in the registry, other important variables such as a complete cardiovascular risk evaluation, other received treatments apart from immunosuppressants and immunoglobulins, and complete lymphocyte subpopulation counts, among others, were not included. Noteworthy, only adult individuals were included, and many hospitals were not reference institutions for treating PIDs. This fact might have influenced the follow-up of CVID patients but is a clear reflection of the Spanish reality in the diagnosis, management, and treatment of this heterogeneous disease. Finally, this study was conceived from a descriptive approach, and no inferential analyses were performed. Data from the GTEM-SEMI-CVID Registry will be used for the design of further studies aiming to characterize and compare patient subpopulations.

## Conclusion

This is the first nationwide multicentric study on CVID patients in Spain including both secondary and tertiary referral hospitals. The data provided by this work stress the need for developing national guidelines to optimize the diagnosis and management of these patients. Chronic dysimmune complications have emerged as the main cause of morbidity and mortality after the introduction of IgRT and pose a great challenge for clinicians treating PIDs. The lack of effective treatments and the use of immunosuppressants for the management of these disorders emphasizes the importance of basic and translational research delving into the pathophysiological pathways involved in immune dysregulation and diffuse lymphocytic infiltration. This would reveal new tailored strategies to reduce immune complications, the associated healthcare burden, and ensure a better quality of life for CVID patients. Moreover, evidence on cardiovascular disease in CVID is lacking and new studies should be designed to address this considerable knowledge gap. Finally, further studies with a large sample size in other regions, and meta-analyses of current evidence are needed to improve the epidemiological knowledge of this complex and heterogeneous disorder.

## Data availability statement

The datasets presented in this article are not readily available because they belong to an institutional multicentric registry from the Spanish Society of Internal Medicine. Requests to access the datasets should be directed to the promotor of the registry, PMM: moral_ped@gva.es.

## Ethics statement

This study has been independently approved by the ethics comittees of all participating institutions. Written informed consent for participation was not required for this study in accordance with the national legislation and the institutional requirements. All authors contributed to the article and approved the submitted version.

## Author contributions

MC-N, VG-B, and PMM conceived the idea, searched the bibliographic materials, and reviewed the existing literature. MC-N, VG-B, and PMM developed the tables. VG-B performed the statistical analysis. MC-N, VG-B, MN-B and PMM contributed to the writing of the article. The rest of the authors contributed in data inclusion and variable collection in their respective centers. All authors are responsible for the care of the patients and provided their data to the GTEM-SEMI-CVID Registry.

## Funding

The article processing charge for publication has been funded by CSL Behring. The funder was not involved in the study design, collection, analysis, interpretation of data, the writing of this article or the decision to submit it for publication.

## Acknowledgments

We would like to thank the Working Group of Minority Diseases of the Spanish Society of Internal Medicine (GTEM), the Spanish Society of Internal Medicine (SEMI) and the Valencian Community Society of Internal Medicine (SMICV) for their support.

## Conflict of interest

The authors declare that the research was conducted in the absence of any commercial or financial relationships that could be construed as a potential conflict of interest.

## Publisher’s note

All claims expressed in this article are solely those of the authors and do not necessarily represent those of their affiliated organizations, or those of the publisher, the editors and the reviewers. Any product that may be evaluated in this article, or claim that may be made by its manufacturer, is not guaranteed or endorsed by the publisher.
