# Supplementary material for: Current clinical spectrum of common variable immunodeficiency in Spain: The multicentric nationwide GTEM-SEMI-CVID registry
Source: Front Immunol. 2022 Oct 28;13:1033666. doi: 10.3389/fimmu.2022.1033666 (PMC9650514; doi:10.3389/fimmu.2022.1033666)
Supplement: Supplementary file 1 [file DataSheet_1.docx]

**Supplementary material**

**Table S1**. Comorbidities of patients who had received or were under corticosteroid treatment

| **Total number of patients who had received or were under corticosteroid treatment** | **N=81** |
| --- | --- |
| Autoimmune cytopenia | N=52 |
| Enteropathy | N=24 |
| GLILD | N=14 |
| NHL | N=9 |
| Ankylosing spondylitis | N=4 |
| Demyelinating CNS disease | N=2 |
| Transverse myelitis | N=2 |
| Optic neuritis | N=1 |
| SLE | N=1 |
| Peripheral neuropathy | N=1 |
| Sjögren disease | N=1 |

CNS, Central Nervous System; GLILD, Granulomatous-Lymphocytic Interstitial Lung Disease; N, number of patients who had received or were under the specified treatment; NHL, Non-Hodgkin lymphoma; SLE, Systemic Lupus Erythematosus

**Table S2**. Comorbidities of patients who had received or were under azathioprine treatment.

| **Total number of patients who had received or were under azathioprine treatment** | **N=29** |
| --- | --- |
| Autoimmune cytopenia | N=21 |
| GLILD | N=12 |
| Enteropathy | N=8 |
| NHL | N=4 |
| Transverse myelitis | N=1 |
| Ankylosing spondylitis | N=1 |
| Vasculitis | N=1 |

GLILD, Granulomatous-Lymphocytic Interstitial Lung Disease; N, number of patients who had received or were under the specified treatment; NHL, Non-Hodgkin lymphoma

**Table S3**. Comorbidities of patients who had received or were under rituximab treatment

| **Total number of patients who had received or were under rituximab treatment** | **N=29** |
| --- | --- |
| Autoimmune cytopenia | N=24 |
| GLILD | N=14 |
| Enteropathy | N=4 |
| NHL | N=6 |
| Transverse myelitis | N=1 |
| Optic neuritis | N=1 |
| Demyelinating CNS disease | N=1 |

CNS, Central Nervous System; GLILD, Granulomatous-Lymphocytic Interstitial Lung Disease; N, number of patients who had received or were under the specified treatment; NHL, Non-Hodgkin lymphoma

**Table S4**. Comorbidities of patients who had received or were under corticosteroids, rituximab, and azathioprine

| **Total number of patients who had received or were under corticosteroids, azathioprine, and rituximab treatment** | **N=17** |
| --- | --- |
| GLILD + autoimmune cytopenia | N=10 |
| GLILD | N=1 |
| Enteropathy | N=3 |
| Autoimmune cytopenia | N=2 |
| NHL | N=3 |
| Transverse myelitis | N=1 |

GLILD, Granulomatous-Lymphocytic Interstitial Lung Disease; N, number of patients who had received or were under the specified treatment; NHL, Non-Hodgkin lymphoma

**Table S5**. Comorbidities of patients who had received or were under mycophenolate mofetil treatment

| **Total number of patients who had received or were under mycophenolate mofetil treatment** | **N=9** |
| --- | --- |
| Autoimmune cytopenia | N=4 |
| GLILD | N=4 |
| Enteropathy | N=2 |

GLILD, Granulomatous-Lymphocytic Interstitial Lung Disease; N, number of patients who had received or were under the specified treatment.

**Table S6**. Comorbidities of patients who had received or were under tacrolimus treatment

| **Total number of patients who had received or were under tacrolimus treatment** | **N=7** |
| --- | --- |
| Autoimmune cytopenia | N=2 |
| GLILD | N=1 |
| Enteropathy | N=3 |
| Demyelinating CNS disease | N=1 |
| Transverse myelitis | N=1 |
| Optic neuritis | N=1 |

CNS, Central Nervous System; GLILD, Granulomatous-Lymphocytic Interstitial Lung Disease; N, number of patients who had received or were under the specified treatment; NHL, Non-Hodgkin lymphoma

**Table S7**. Comorbidities of patients who had received or were under antimalarial treatment

| **Total number of patients who had received or were under antimalarial treatment** | **N=6** |
| --- | --- |
| Autoimmune cytopenia | N=5 |
| SLE + autoimmune cytopenia | N=2 |
| SLE | N=1 |

N, number of patients who had received or were under the specified treatment; SLE, Systemic Lupus Erythematosus.
